# Hounsfield unit value on CT as a predictor of cage subsidence following stand-alone oblique lumbar interbody fusion for the treatment of degenerative lumbar diseases

**DOI:** 10.1186/s12891-021-04833-1

**Published:** 2021-11-17

**Authors:** Jing Zhou, Chao Yuan, Chao Liu, Lei Zhou, Jian Wang

**Affiliations:** Department of Orthopaedic Surgery, Affiliated Xinqiao Hospital, Army Medical University, No.183, Xinqiao Street, Shapingba District, Chongqing, 400037 People’s Republic of China

**Keywords:** Oblique lumbar interbody fusion, Hounsfield units, Osteoporosis, Cage subsidence, Degenerative lumbar diseases

## Abstract

**Background:**

To investigate the correlation between vertebral Hounsfield unit (HU) values and cage subsidence in patients treated with stand-alone (SA) OLIF.

**Methods:**

A retrospective review of collected data was performed on 76 patients who underwent SA OLIF. We utilized the HU value for lumbar bone mineral density (BMD) obtained on preoperative CT. The vertebral HU values of patients with subsidence were compared to those without subsidence. The correlation between cage subsidence and clinical score was investigated.

**Results:**

Sixteen patients (21.1%) had at least radiographic evidence of interbody cage subsidence. The average cage subsidence was 2.5 ± 1.3 mm (range 0.9-4.8 mm). There were no significant differences in sex, BMI, preoperative diagnoses, or fused level (*p* > 0.05); however, there were significant differences between the cage subsidence group and the nonsubsidence group in age, average of the lowest T-score, and average HU value, including for the L1 vertebrae, L1-L4 horizontal plane, and L1-L4 sagittal plane (*p* < 0.05). The average HU value of the L1-L4 horizontal plane showed a more predictable AUC of 0.909 (95% CI, 0.834–0.984; *P* < 0.001) compared with the average of the lowest T-score following an AUC of 0.791 (95% CI, 0.674–0.909; *P* < 0.001). Based on logistic regression analysis, the average HU value of the L1-L4 horizontal plane (OR, 0.912; 95% CI, 0.861–0.966; *P* = 0.002) was an independent factor influencing cage subsidence.

**Conclusions:**

Patients with lower average HU values of the lumbar vertebrae are at a much higher risk of developing cage subsidence after SA OLIF. Measurement of preoperative HU values on preexisting CT scans could be rapid, simple and feasible.

## Background

Recently, oblique lumbar interbody fusion (OLIF) has been introduced, providing a novel corridor to access the lumbar disc space [1, 2]. This approach uses the anatomic space between the anterior vessels and psoas muscles, allowing for efficient clearance of disc space and application of a large interbody cage to afford distraction for indirect decompression and endplate preparation for fusion. The OLIF technique is increasingly employed to treat structural degenerative conditions of the lumbar spine. Although OLIF combined with bilateral pedicle screw fixation is a widely performed procedure that provides a variety of advantages, such as excellent fixation intensity and a high fusion rate, it can be used as a stand-alone procedure to manage isolated degenerative disc diseases or spondylolisthesis [3–5]. More recently, it has been used as a part of reconstructive surgery in cases of degenerative deformity therapy [6]. Stand-alone OLIF (SA OLIF) has become a popular method of treatment of lumbar degenerative disease. Indirect decompression of the neural constructures is obtained by the restoration of the disk height and the reduction of slipped vertebra with the insertion of a proportionally sized cage. Additional posterior instrumentation has been suspected to result in more extensive dissection and blood loss, longer duration of surgery, higher risk of implant-related complications, and greater medical costs.

Cage subsidence is a potentially devastating complication after lumbar interbody fusion and is also an important issue in SA OLIF procedures. The development of cage subsidence is presumed to be a multifactorial process. Several potential risk factors have been examined in the literature, including overdistraction, insufficient cage width, poor construct length, lateral plating, and endplate violation [7, 8]. Bone quality is believed to be one of the important factors that cause cage subsidence associated with the lumbar interbody. Dual-energy X-ray absorptiometry (DXA) is cost effective, widely used clinically to diagnosis osteoporosis and represents the current gold standard for bone mineral density (BMD) assessment. However, various limitations of DXA have been described, such as distortion of estimated bone mineral mass values caused by overlying soft tissue, vascular calcifications, bowel content and degenerative spine changes. BMD measurement by vertebral body Hounsfield units (HU) as a predictor of cage subsidence should be included in preoperative planning for surgeons to determine surgical options. To date, few studies have attempted to assess BMD and cage subsidence following SA OLIF for degenerative lumbar diseases. The purposes of this work were to assess the relationship between BMD and cage subsidence following SA OLIF and to determine whether the vertebral body HU value should influence the operative plan in patients undergoing SA OLIF.

## Materials and methods

### Patients

After obtaining our institutional review board approval, a retrospective review of collected data was performed on all patients who underwent SA OLIF at a single institution between February 2015 and April 2020. All patients were followed up with 28.2 ± 9.3 months. The inclusion criteria for this study were as follows: (1) degenerative spondylolisthesis grade 1° according to the Meyerding classification; (2) lumbar instability; (3) discogenic back pain; (4) adjacent segmental diseases; (5) mild adult degenerative scoliosis; (6) chronic lower back pain and/or leg pain unresponsive to conservative therapy for at least 6 weeks; and (7) no experience of endplate damage during the procedure. The exclusion criteria for the study were as follows: (1) lumbar spondylolisthesis grade > 1°; (2) lumbar spondylolysis; (3) spinal infection; (4) spinal tumor pathologies; (5) spinal trauma; and (6) diseases involving the L5–S1 vertebrae.

All of the patients included had preoperative evaluations with detailed neurologic examination and radiologic imaging, which involved static (anterior-posterior and lateral) and dynamic (flexion and extension) plain lumbar radiographs, magnetic resonance imaging (MRI), and computed tomography (CT) scans.

Preoperative CT was routinely used to calculate HU values. The vertebral body HU values were measured according to the technique described by Schreiber [9]. Using standard picture archiving and communication system (PACS) software, an elliptical area of interest (AOI) was drawn on an axial image of the lumbar vertebral body, including the largest possible cancellous bone and excluding cortical edges, osteophytes, and osseous abnormalities, such as apparent sclerotic areas. Special attention was paid not to allow the AOI to include intervertebral spaces, which might cause inaccurate measurement of trabecular HU values in the vertebral body. Three measurements were obtained: just superior to the inferior endplate; the mid-vertebral body; and just inferior to the superior endplate. The picture system calculates the HU value of the area within the ellipse. The vertebral body BMD was defined as the average HU value of the three AOIs. A mid-sagittal CT image of the lumbar spine provided measurement of HU values. One major challenge of endplate CT assessments is the reproducibility of measurements because the methodology is not as standardized as a routine assessment. Therefore, the vertebral body HU values were measured in this series. DXA scans were performed routinely. The lowest T-score of the hips and lumbar spine was recorded based on preoperative DXA scans because the criterion for osteoporosis was the lowest T-score ≤ − 2.5 [10]. Disc height was measured on the midsagittal image of CT scans at the midway point of the vertebral bodies.

The average patient age at the time of surgery was 56.1 ± 10.4 years old (29–81 yr), and the study group comprised 46 women and 30 men. The most frequent diagnosis was degenerative spondylolisthesis grade 1° (32 cases, 42.1%), followed by lumbar instability (17 cases, 22.4%), adjacent segmental disease (18 cases, 23.7%), adult degenerative scoliosis (6 cases, 7.9%) and discogenic low back pain (3 cases, 3.9%). A total of 84 levels were treated: 92.1% one level, 5.3% two level, and 2.6% three level surgeries. The patients’ demographic characteristics are listed in Table 1.

**Table 1:** Demographics and treatment data for 76 patients

Characteristics	All (*n* = 76)
Number of patients	76
Mean age (years)	56.1 ± 10.4
BMI (kg/m^2^)	24.7 ± 2.2
Sex (M/F) (% male)	30/46 (39.5)
Diagnoses [no.(%) of patients]	
Degenerative spondylolisthesis	32 (42.1)
Lumbar instability	17 (22.4)
Adjacent segmental disease	18 (23.7)
Adult degenerative scoliosis	6 (7.9)
Discogenic back pain	3 (3.9)
Fused level [no.(%)of patients]	
One level	70 (92.1)
Two level	4 (5.3)
Three level	2 (2.6)
Operative segment	
L2-L3	4 (4.8)
L3-L4	24 (28.6)
L4-L5	56 (66.7)

*Abbreviations*: *BMI* Body Mass Index

### Surgical technique

All of the patients underwent OLIF only through a left-side approach without neurophysiologic monitoring. The procedures were performed utilizing expandable retractors (OLIF 25 system; Medtronic, Memphis, TN, USA) according to the procedure described previously [3]. Polyetheretherketone (PEEK) intervertebral cages (OLIF25 Clydesdale Spinal System; Medtronic SofamorDanek, MN, USA) filled with allograft bone were used to achieve fusion. Special care was taken to avoid endplate fracturing during the disc space preparation and to span the apophyseal rings of both end plates during cage insertion. No supplementary anterior or posterior instrumentation or direct decompression was performed for 76 patients.

### Clinical and radiographic measures

Clinical parameters included visual analog scores (VAS) and the Oswestry Disability Index (ODI). The ODI, version 2.0, was used both before surgery and after surgery. Considering that the sex question (Section 8) was omitted, the total possible score was 45. After discharge from the hospital, the patients had regular follow-ups conducted by the corresponding author. Plain lumbar radiographs and, if uncertain, computed tomography scans were ordered to detect cage subsidence. Cage subsidence was defined as cage vertical protrusion through the cephalad or caudal (or both) endplate of the vertebral body. Subsidence was measured by gauging the maximal migration of the cage into the endplate on lateral images of standing lumbar radiographs at a minimum of 6 months postoperatively. If a patient had multiple radiographs during the period, the last radiograph was used for subsidence assessment. Cage subsidence was assessed by two researchers after a training session and under the supervision of a board-certified orthopedic spine surgeon. Furthermore, we evaluated the correlation between cage subsidence and clinical scores.

Interbody fusion was determined by lumbar radiographic examination and/or CT scans if needed at the final follow-up. The criterion for pseudarthrosis was the presence of regional motion of more than 3° or intervertebral translation of more than 3 mm on lateral dynamic X-ray images. The criterion of fusion status from the CT scan was the presence of bony bridge in the sagittal and coronal reconstruction planes and its connections to the lower and upper endplates. If there were any defects in any position, the fusion status was classified as pseuarthrosis.

### Statistical analyses

The values are shown as the mean ± SD. Statistical analyses were performed using SPSS software (version 23, USA). *P* values < 0.05 were accepted for significance. The Shapiro-Wilk test was used to verify the normal distribution of continuous variables. The independent sample t-test was used for variables that followed a normal distribution. The Mann-Whitney U test was used for those not following normal distribution. The paired t-test was used for intragroup comparison. The chi-square test or Fisher’s exact test was used for categorical data. Intra-class correlation coefcient (ICC) was used to evaluate inter-observer and intra-observer reliability of HU and cage subsidence measurements. (ICC ≥ 0.8 was considered to indicate excellent reliability.) Receiver operating characteristic (ROC) curves were used to establish a separation criterion between the two groups. The areas under the ROC curve (AUCs) were calculated for regional assessment. Logistic regression analysis was used to identify the independent factors of cage subsidence, and the results are presented as odds ratios (ORs) with 95% confidence intervals. Spearman’s correlation analysis was used to identify the correlation between the distance of cage subsidence and improvement in pain VAS or ODI scores.

## Results

A total of 84 segments in 76 patients were included in the final analysis (30 male and 46 female patients; mean age 56.1 ± 10.4 years old), with a mean BMI of 24.7 ± 2.2 kg/m^2^. The patients were divided into a cage subsidence group (*n* = 16) and a nonsubsidence group (*n* = 60) based on the occurrence of cage subsidence.

### HU value and cage subsidence

The intra-observer and inter-observer reliability in measuring HU value was excellent with ICCs of 0.989 and 0.972, respectively. The ICCs of the intra-observer and inter-observer reliability were 0.986 and 0.963 in measuring the distance of cage subsidence. Sixteen of 76 (21.1%) patients developed at least radiographic evidence of subsidence. The mean subsidence measured by the last follow-up lateral radiographs was 2.5 ± 1.3 mm (range, 0.9-4.8 mm). Cage subsidence of < 2 mm was found in 9 patients, and subsidence of ≥2 mm was found in 7 patients. The comparison of demographics and clinical data between the cage subsidence and nonsubsidence groups is shown in Table 2. The patients with cage subsidence of ≥2 mm had significantly lower HU values on the L1-L4 horizontal plane (81.2 ± 10.4) than the patients with subsidence of < 2 mm (106.5 ± 13.7) (*P* = 0.007). There were no significant differences in sex, BMI, diagnoses, or fused level (*p* > 0.05); however, there were significant differences between the two groups in age, lowest T-score, and HU value, including L1 vertebrae, L1-L4 horizontal plane, and L1-L4 sagittal plane (*p* < 0.05). We recorded the disc heights at both the operating and suprajacent levels in each patient. The preoperative and postoperative disc heights showed no significant difference between the two groups (*p* > 0.05) (Table 3).

**Table 2:** Comparison of demographic and clinical data between the two groups

	Subsidence group *(n* = 16)	Nonsubsidence group (*n* = 60)	*P* value
Mean age (years)	64.4 ± 7.1	54.0 ± 10.1	< 0.001
Sex (M/F) (% male)	6/10 (37.5)	24/36 (40.0)	0.856
BMI (kg/m^2^)	24.6 ± 1.8	24.7 ± 2.3	0.892
Lowest T-score	− 2.8 ± 0.8	− 1.6 ± 1.3	< 0.001
HU value			
L1 vertebrae	98.5 ± 18.8	140.8 ± 28.6	< 0.001
L1-L4 horizontal plane	95.4 ± 17.6	136.8 ± 28.3	< 0.001
L1-L4 sagittal plane	90.7 ± 22.4	135.2 ± 31.3	< 0.001
Operated segment	96.3 ± 19.2	134.4 ± 29.7	< 0.001
Upper vertebrae of operated segment	94.7 ± 20.4	133.2 ± 28.8	< 0.001
Lower vertebrae of operated segment	97.8 ± 20.5	135.6 ± 32.4	< 0.001
Diagnoses [no.(%) of patients]			
Degenerative spondylolisthesis	8 (50)	24 (40)	0.472
Lumbar instability	5 (31.3)	12 (20)	0.534
Adjacent segmental disease	3 (18.8)	15 (25)	0.848
Adult degenerative scoliosis	0 (0)	6 (10.0)	0.426
Discogenic back pain	0 (0)	3 (5.0)	1.000
Fused level [no.(%)of patients]			
One level	16 (100)	54 (90)	0.426
Two level	0 (0)	4 (6.7)	0.573
Three level	0 (0)	2 (3.3)	1.000

*Abbreviations*: *BMI* Body Mass Index

**Table 3:** Comparison of disc heights between the two groups

	Subsidence group (*n* = 16)	Nonsubsidence group (*n* = 60)	*P* value
Preoperative operative levels	9.0 ± 1.5	8.8 ± 1.5	0.463
Preoperative suprajacent levels	10.2 ± 1.2	10.3 ± 1.4	0.794
Immediate postoperative operative levels	11.9 ± 0.9	11.7 ± 1.4	0.541

**Fig. 1:**
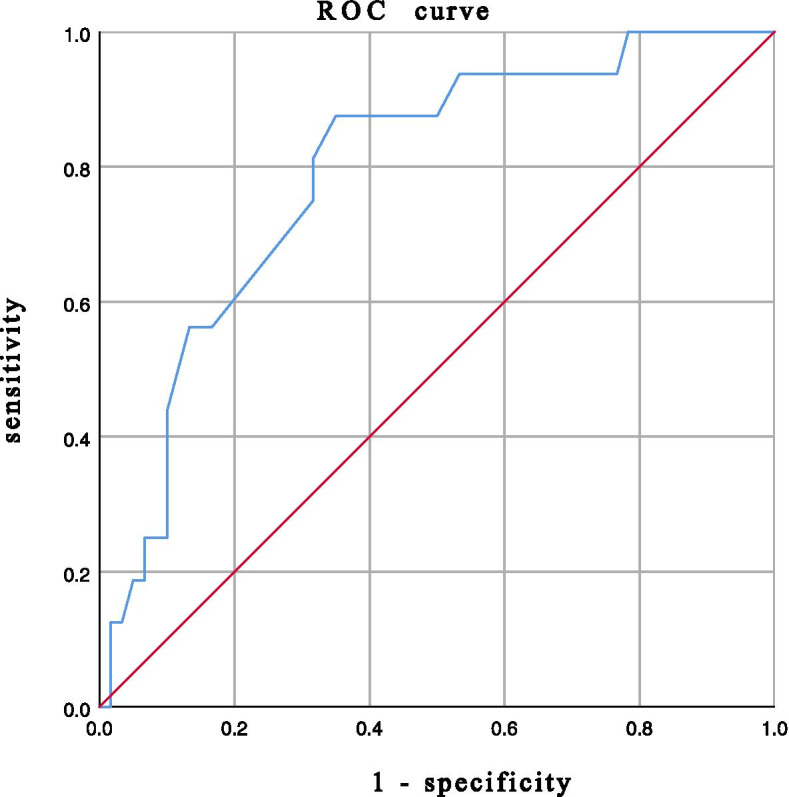
Receiver operating characteristic curve for the lowest T-score as a predictor of cage subsidence, with an area under the curve of 0.791, sensitivity of 87.5% and specificity of 65.0%

**Fig. 2:**
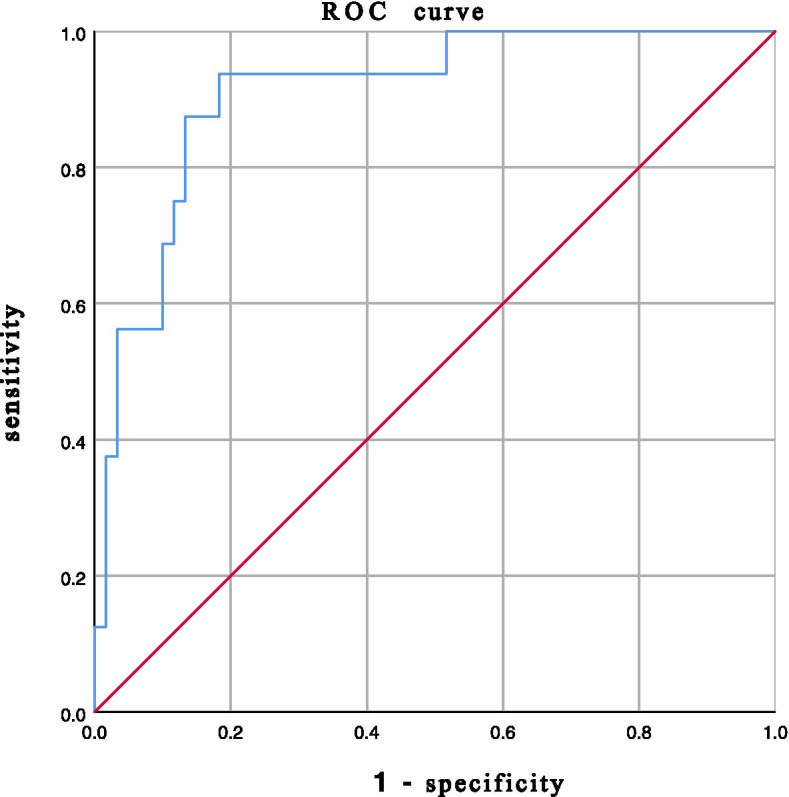
Receiver operating characteristic curve for Hounsfield units of the L1-L4 horizontal plane as a predictor of cage subsidence, with an area under the curve of 0.909, sensitivity of 93.8% and specificity of 81.7%

On the basis of ROC analysis (Table 4), the lowest T-score had a significant ability to predict cage subsidence, with an AUC of 0.791 (95% CI, 0.674–0.909; *P* < 0.001) (Fig. 1), while the average HU value of the L1-L4 horizontal plane followed a more predictable AUC of 0.909 (95% CI, 0.834–0.984; *P* < 0.001) (Fig. 2). The average HU value of 115.7 on the L1-L4 horizontal plane with balanced sensitivity (93.8%) and specificity (81.7%) in predicting cage subsidence was chosen as the cutoff value for finding patients with a higher risk of subsidence. The cutoff value of the lowest T-score was − 2.55 with balanced sensitivity (87.5%) and specificity (65.0%) (*P* < 0.001).

**Table 4:** Results of ROC analysis

	AUC (95%CI)	*P* value	Cutoff	Sensitivity	Specificity
Average of the lowest T-score	0.791 (0.674-0.909)	< 0.001	−2.55	87.5%	65%
Average HU value					
L1	0.907 (0.840-0.975)	< 0.001	124.6	93.8%	78.3%
L1-L4 horizontal plane	0.909 (0.834-0.984)	< 0.001	115.7	93.8%	81.7%
L1-L4 sagittal plane	0.891 (0.809-0.973)	< 0.001	114.9	87.5%	81.7%
Operated segment	0.880 (0.789-0.970)	< 0.001	116.1	93.8%	80.0%
Upper vertebrae of operated segment	0.885 (0.785-0.986)	< 0.001	111.8	93.8%	81.7%
Lower vertebrae of operated segment	0.866 (0.782-0.949)	< 0.001	121.0	93.8%	73.3%

*Abbreviations*: *ROC* Receiver operating characteristic curve

The factors with a *P* value of < 0.1 in Table 2 were chosen as potential cage subsidence influencing factors and entered into the logistic regression, including age, the average HU value of the L1-L4 horizontal plane with the highest AUC, and the lowest T-score. The other average HU values were not included in the logistic regression because they were highly correlated with the average HU value of the L1-L4 horizontal plane. Further logistic regression analysis showed that the average HU value of the L1-L4 horizontal plane (OR, 0.912; 95% CI 0.861–0.966; *P* = 0.002) was an independent factor influencing cage subsidence. The OR of the lowest T-score was 1.302 (95% CI, 0.534–3.179; *P* = 0.562), and the OR of age was 1.017 (95% CI, 0.908-1.139; *P* = 0.770).

### Clinical outcomes

A total of 76 patients, including 84 segments, were available for review. Both the ODI and VAS scores significantly decreased at the last follow-up compared to the preoperative scores, not only in the subsidence group but also in the nonsubsidence group (*P* < 0.05). However, the preoperative and postoperative VAS and ODI scores showed no significant differences between the two groups (*P* > 0.05) (Table 5).

**Table 5:** Comparison of clinical outcomes between the subsidence group and the nonsubsidence group

	Clinical outcomes	Subsidence group	Nonsubsidence group	*P* value
Preoperatively	VAS (Back)	5.4 ± 0.8	5.3 ± 0.9	0.615
	VAS (Leg)	6.4 ± 1.0	6.1 ± 0.7	0.086
	ODI	27.4 ± 4.2	27.3 ± 5.7	0.614
Postoperative at 3 months	VAS (Back)	2.3 ± 0.7	2.0 ± 0.6	0.108
	VAS (Leg)	2.9 ± 0.6	2.6 ± 0.5	0.155
	ODI	15.4 ± 2.6	14.2 ± 2.8	0.071
Postoperative at 6 months	VAS (Back)	1.9 ± 0.6	1.6 ± 0.7	0.123
	VAS (Leg)	2.1 ± 0.6	1.9 ± 0.6	0.206
	ODI	13.5 ± 2.3	12.7 ± 2.8	0.228
Last follow-up	VAS (Back)	1.4 ± 0.5	1.3 ± 0.7	0.473
	VAS (Leg)	1.9 ± 0.4	1.8 ± 0.6	0.196
	ODI	12.2 ± 2.4	12.0 ± 3.0	0.723

*Abbreviations*: *VAS* Visual Analog Scale, *ODI* Oswestry Disability Index

Of the 16 patients with cage subsidence, 7 patients suffered from debilitating back pain within 3 months after SA OLIF surgery. After these patients wore a waist brace and underwent conservative treatment, the symptoms of low back pain were significantly reduced, and no patients required revision surgery. No recurrent lower extremity radiculopathy occurred. Transient symptoms, including left groin and/or thigh dysesthesia, in six patients disappeared within 2 months after conservative therapy. When comparing the clinical score of last follow-up between cage subsidence of < 2 mm group and subsidence of ≥2 mm group,there were no significant differences in VAS back pain scores(1.6 ± 0.5 VS 1.3 ± 0.5 *P* = 0.296), VAS leg pain scores (2.0 ± 0.5 VS 1.9 ± 0.4 *P* = 0.534), or ODI scores (11.8 ± 2.8 VS 12.7 ± 2.0 *P* = 0.463). The distance of cage subsidence at the last follow-up showed no significant correlation with improvements in pain VAS (r_s_ = − 0.123, *P* = 0.649) or ODI scores (r_s_ = 0.438, *P* = 0.09).

### Fusion rates

The overall fusion rate was 97.4% in our series. At the final follow-up, nonfusion status was observed in one patient with cage subsidence of < 2 mm. A higher fusion rate of 98.3% was found in the nonsubsidence group compared to the subsidence group (93.8%). However, there was no significant difference between the groups (*P* = 0.379).

## Discussion

Previous studies demonstrated that osteoporosis is one of the major risk factors for cage subsidence after lumbar interbody fusion. In patients with poor bone quality, consideration could be made to supplement the lateral lumbar interbody fusion cage with posterior instrumentation [11]. In a large series of patients, Le et al [7] reported a subsidence rate of 14.3%, while Marchi et al [8] reported a subsidence rate of 10-30% depending on graft size. The development of cage subsidence is presumed to be a multifactorial process. Several potential risk factors have been examined in the literature, consisting of overdistraction, smaller cage width, construct length, lateral plating, and endplate violation, which are directly related to surgical technique and intraoperative decision making [7, 8, 12–14].

Patients with cage subsidence might suffer from axial pain and recurrent neurological symptoms, although SA OLIF could be as effective as OLIF aided by different fixation devices with less blood loss, shorter operative time, and lower operative costs for implants. The contact area between the cage and the endplate would most likely be directly related to cage width, which in turn would be associated with cage subsidence. Larger diameter cages have been shown to have a lower risk of cage subsidence. Therefore, theoretically, the use of a larger cage in the SA OLIF procedure would improve spinal stabilization and decrease the likelihood of cage subsidence. Low bone mineral density (BMD) has been previously tied to higher rates of postoperative interbody cage subsidence in patients undergoing OLIF. Osteoporosis could play a more important role in the occurrence of cage subsidence during SA-OLIF than cage size. For this reason, accurate preoperative assessment of a patient’s underlying bone quality in the operated vertebral body is paramount for OLIF, especially SA OLIF.

Dual-energy X-ray absorptiometry (DXA) is the current “gold standard” to assess BMD and is widely used clinically to diagnose osteopenia or osteoporosis. However, a growing body of research literature has suggested that in vivo assessments of BMD using DXA are inaccurate and imperfect. Alternative means of preoperative bone quality assessment have been investigated for patients being considered for spinal fusion [9, 15, 16]. The HU value is less affected by lumbar degenerative conditions than DXA by avoiding cortical bone, vascular calcification, and degenerative structures. Considering that lumbar CT is a routine preoperative examination for patients requiring lumbar fusion, the HU value can be measured on preexisting CT images at no additional cost and without radiation. Another advantage of a CT-based technique over DXA is that CT-based techniques can selectively measure detailed site-specific bone density, such as the pedicles and endplates [17].

The BMD of the endplate region was highly reliable and more predictive of vertebral fracture and cage subsidence [18]. Modic changes and endplate sclerosis with higher HU values are potential predictors for preventing cage subsidence, making the endplate with MCs or sclerosis a potentially suitable condition that can be submitted to SA OLIF [19]. Microstructural and/or material property changes associated with modic type 2 changes, which are independent of regional endplate BMD values, however, might have a protective effect on cage subsidence after stand-alone OLIF [20]. However, few studies have comprehensively evaluated the specific relationship between vertebral body HU and interbody graft failure during SA OLIF. It is worth assessing the influence of site-specific BMD on the risk of cage subsidence since the contact surfaces between cage and bone only include the implant and endplates in SA OLIF. However, it is difficult to determine the area of interest (AOI) and measure the HU value of the endplate because of its irregular shape. Therefore, the mean HU value of the three AOIs was measured as the vertebral body HU value to investigate the correlation between BMD and cage subsidence in our series.

According to a systematic review of studies that specified the subsidence rate, the pooled patient population equaled 1362 patients, 141 of whom experienced cage settling after LLIF. Therefore, the estimated incidence of subsidence reached 10.3% [21]. The present study demonstrated that there was a relatively high rate of cage subsidence (21.1%) in 76 patients compared to that (18.7%) reported by Abe et al. [22] There were significantly lower vertebral body HU values in 16 patients with subsidence than in 60 patients without subsidence. Our data demonstrated that the average HU value of the L1-L4 horizontal plane was more predictive of cage subsidence, with an AUC of 0.909 (95% CI, 0.834–0.984; *P* < 0.001), than DXA t-scores, with an AUC of 0.791 (95% CI, 0.674–0.909 *P* < 0.001). Unlike DXA t-scores, accepted standard cutoff values for HU measurement have not been determined. Using 115.7 HU in the L1-L4 horizontal plane as the cutoff value provided 93.8% sensitivity and 81.7% specificity in our study. Patients with HU values less than 115.7 who underwent stand-alone OLIF were at greater risk for developing cage subsidence. Our findings suggested that a significant association exists between lower HU values of the lumbar vertebral body and the likelihood of cage subsidence after SA OLIF. Therefore, we recommend routinely measuring the HU value on preoperative CT scans when planning SA OLIF for lumbar degenerative conditions. If spine surgeons decide to perform SA OLIF and avoid the occurrence of cage subsidence, patients with HU values greater than 110 should be chosen as candidates.

Considering that no apparent loss of postoperative disc height was observed when local and mild-scale cage migration in the endplates occurred, we did not use the grading system of Marchi et al. [8] Our results demonstrated that patients with cage subsidence of ≥2 mm had a significantly lower average HU value in the lumbar spine than the patients with subsidence of < 2 mm, which was comparable to that reported by Oh et al. [23]

The clinical outcomes were comparable between the subsidence group and the nonsubsidence group, although 16 cases (21.1%) of cage subsidence were found in this study. We recommend a longer time for the patient to wear the waist brace to prevent further subsidence. It is questionable whether mild or moderate cage subsidence is clinically significant. Cho et al [24] concluded that cage subsidence did not significantly affect clinical outcomes. Tempel et al [25] reported that severe cage subsidence was significantly associated with revision surgery after SA-OLIF, in contrast to Cho et al.

Our patient population was relatively heterogeneous with regard to surgical indication, which included spondylolisthesis, lumbar instability, kyphoscoliosis, adjacent segmental diseases, and discogenic pain, despite the patients undergoing the same surgical procedure. There were other limitations, such as the relatively small sample size, short follow-up time, and retrospective nature of this study. Additionally, we were not able to definitively determine endplate injury during endplate preparation and cage insertion, which might have played a role in cage subsidence. We did not investigate the correlations among cage size, cage positioning, excessive disc space correction and subsidence. Finally, we did not evaluate any preventive measures for cage subsidence.

## Conclusion

In summary, SA OLIF has many advantages that render it attractive; however, there is a risk of cage subsidence. Patients with lower vertebral body HU values are at significantly higher risk of experiencing cage subsidence. Accurate assessment of preoperative HU values on preexisting CT scans is rapid, simple and reliable when planning the procedure. SA OLIF should be performed in patients with good bone quality. However, our results showed that cage subsidence is not related to clinical deterioration.

## Data Availability

The datasets used and/or analysed during the current study are available from the corresponding author on reasonable request.

## Abbreviations

AOI: Area of interest
AUC: Areas under the ROC curve
BMD: Bone mineral density
BMI: Body Mass Index
DXA: Dual-energy x-ray absorptiometry
HU: Hounsfield Units
ODI: Oswestry Disability Index
PACS: Picture archiving and communication system
ROC: Receiver operating characteristic curve
SA OLIF: Stand-alone Oblique Lumbar Interbody Fusion
VAS: Visual Analog Scale
